# Epidemiology and molecular detection of *Anaplasma* spp. in goats from Chattogram district, Bangladesh

**DOI:** 10.1002/vms3.775

**Published:** 2022-02-26

**Authors:** Mizanur Rahman, Md. Rayhan Faruque, Md. Mizanur Rahman, Mohammed Yousuf Elahi Chowdhury

**Affiliations:** ^1^ Faculty of Veterinary Medicine Teaching and Training Pet Hospital and Research Center Chattogram Veterinary and Animal Sciences University Khulshi Bangladesh; ^2^ Faculty of Veterinary Medicine Department of Medicine and Surgery Chattogram Veterinary and Animal Sciences University Khulshi Bangladesh

**Keywords:** *Anaplasma* spp, goat, molecular detection, prevalence, risk factors

## Abstract

**Objectives:**

Anaplasmosis is an economically important disease affecting cattle, buffalo, sheep, goat etc. The study was conducted to determine the prevalence, potential risk factors and molecular identification of circulating *Anaplasma* spp. in goats in Chattogram district, Bangladesh.

**Material and methods:**

Four hundred blood samples were collected from goats of different ages, breeds, sex, coat color and body condition. These goats were selected based on some inclusion criteria through the period of July 2017 to June 2018. Samples were examined microscopically (Giemsa staining method) followed by polymerase chain reaction (PCR) and sequencing to identify of *Anaplasma* spp.

**Results:**

The overall prevalences were estimated 5.75% (23/400) and 15.75% (63/400) by microscopy and PCR, respectively. *Anaplasma ovis (A. ovis*) and *Anaplasma marginale* (*A. marginale*) were identified with the prevalence of 14.75% (59/400) and 1.0% (4/400), respectively through PCR. Among different risk factors, jamnapari breed (*p* = 0.027), no use of acaricide (*p* = 0.025) and presence of tick (*p* < 0.01) were found to be significantly associated with anaplasmosis. Sequence analysis of msp4 gene revealed that, *Anaplasma* spp. detected in the present study were highly similar with those of China, Venezuela, Mongolia, Spain, Tunisia, Cyprus, Italy, Brazil, Argentina, Australia, Japan and Columbia.

**Conclusions:**

In conclusion, strategic use of acaricide can control tick that ultimately will control the anaplasmosis in goats. Besides, rearing local goats in compare to cross and exotic breed are also recommended for the farmer to prevent the disease.

## Graphical abstract

The overall prevalence of anaplasmosis in goats was estimated 5.75% and 15.75% by microscopy and PCR, respectively, in this study. *Anaplasma ovis* and *Anaplasma marginale* were identified as 14.75% and 1.0%, respectively, through PCR. Tick infestation on goats and no use of acaricide were identified as significant risk factors for anaplasmosis.

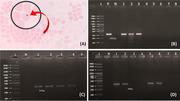

## 1 INTRODUCTION

Goats are one of the important livestock species in rural economy and nutrition with great potentiality to alleviate the poverty of Bangladesh. It is also known as ‘poor man's’ cow as it is generally reared by ultra poor women having little capital investment (Nath et al., 2014). It helps the landless and marginal farmers through women empowerment, youth employment and foreign exchange earnings (Rana, 2015). However, various diseases of goats may cause huge economic losses to the farmer and ultimate the economy of the country through mortality, production losses and addition of extra cost of treatment for sick animals (Singh & Prasad, 2008). Anaplasmosis is among them considering one of the top 10 economically important diseases affecting endemically in tropical, subtropical, and sporadically in temperate region (Rajasokkappan & Selvaraju, 2016). It is a vector borne (tick) disease of ruminants caused by obligate intra‐erythrocytic rickettsial organism of the genus *Anaplasma* and characterised clinically by fever, inappetance, decrease milk yield, progressive anaemia, icterus, brownish urine, pale mucous membrane, labored breathing and constipation (Razmi et al., 2006). Currently, there are six recognised species that have been identified under this genus: *Anaplasma ovis*, *Anaplasma marginale*, *Anaplasma centrale*, *Anaplasma platys*, *Anaplasma bovis* and *Anaplasma phagocytophilum*. Among them, *Anaplasma ovis (A. ovis)* found as most pathogenic and *Anaplasma marginale (A. marginale)* as subclinical form in small ruminants (Razmi et al., 2006).

The climate of Bangladesh is usually hot and humid in nature and the geographical location of Chattogram (study area) is very conducive to a wide variety of ticks that can transmit *Anaplasma* spp. in goats (Ananda et al., 2009; Belal et al., 2014; Kakarsulemankhel, 2011). However, little information is known on caprine anaplasmosis though the bovine anaplasmosis has been extensively studied in this region. In cattle, the prevalence of anaplasmosis has been reported as 70% in Sirajganj, 8.21% in Chattogram, 33% in Baghabari milk shed areas (Belal et al., 2014; Mannan, 2017; Talukdar & Karim, 2001); and in goats 2.48% in Chattogram (Nath et al., 2014). In these studies, diagnosis was done on the basis of clinical signs and Giemsa staining method though these methods of diagnosis have several limitations; for example, the carrier animal may goes unnoticed and differentiation between *Anaplasma* spp. is quite difficult as they are morphologically very similar (Ahmadi‐Hamedani et al., 2009). Thus, polymerace chain reaction (PCR) has become preferred method for the diagnosis of anaplasmosis due to its high sensitivity and specificity than other conventional methods (Lew et al., 2002). Moreover, the msp4 genes involve in interactions with both vertebrate and invertebrate hosts, and evolve more rapidly than other genes are used to identify *Anaplasma* sp. (Ahmadi‐Hamedani et al., 2009). Most importantly, as far our concern, reports on molecular detection, prevalences and associated risk factors of anaplasmosis in goat have not yet been documented in Bangladesh. Therefore, the study was designed to detect the prevalence through the amplification of 16SrRNA and msp4 genes, along with potential risk factors which may help to establish the preventive and control measure of anaplasmosis in goats.

## 2 MATERIAL AND METHODS

### 2.1 Description of study area

The study goats were selected from Teaching Veterinary Hospital located in Chattogram city and regularly receives various patients from different area of Chattogram district. Chattogram is located in south‐eastern part of the Bangladesh. Its area is 5282.98 sq km, located in between 21°54' and 22°59' north latitudes and in between 91°17' and 92°13' east longitudes. It is bounded by Khagrachhari and Rangamati districts and Tripura state of India on the north, Cox's Bazar district on the south, Bandarban, Rangamati and Khagrachari districts on the east and Noakhali district and the Bay of Bengal on the west. It consists of 14 Upazilla (Administrative locations) and Chattogram Metropolitan Area. The tropical monsoon climatic condition characterises by annual average temperature of 13^o^C to 32^o^C, humidity of 70–85% and rainfall of 5.6 mm to 727.0 mm.

### 2.2 Study population and sample collection

The study was conducted from July 2017 to June 2018. The study population was goats that were visited to the Teaching Veterinary Hospital from different area of Chattogram district for the purpose of health check up, vaccination, deworming and treatment of illness. Immediately after registration, clinical history was taken and physical examination was performed for each goat. Physical examination included observation of general appearance, examination of backbone and ribs, observation of visible mucous membrane, palpation of superficial lymhnode, recording of body temperature etc. Goats more than 6 months old that had at least one or more following criteria like presence of vector ticks on the body, fever, anorexia, anaemia, swollen lymphnode and jaundice were considered for sample collection. Based on these criteria, the required numbers of study goats were selected. Sample size (numbers of goats) was determined by the following formula (Thrusfield, 2005).

$$N=\frac{1.96^{2}\times \mathrm{Pexp}\;\left(1-\mathrm{Pexp}\right)}{d^{2}},$$

where *N* is required sample size, Pexp is expected prevalence = 50% = 0.5 (we assumed prevalence was uknown), *d* is desired absolute precision = 5% = 0.05, 1.96 is the value of *z* at 95% confidence interval

Accordingly, the required numbers of goats were 384. However, to increase precision the sample size was increased and total of 400 goats were included in the study.

All relevant data such as breeds (bengal goat, jamnapari, and crossbreed), age category (young, adult and old), sex (male and female), body color (black, brown, gray, white and mixed), body condition score (very thin, thin and good), different management system like rearing system (backyard, intensive and extensive), grazing (group, individual and zero grazing) and flock size (large, medium and small), acaricide using history (yes or no), presence of tick on the body surface (yes or no) and date of registration were recorded using a close ended questionnaire by face to face interview of owner and close examination of goat. After that, blood sample (5 ml) was collected from jugular vein of each goat using sterile disposable needle. Before collection, the puncture area was cleaned and disinfected with 70% alcohol. Thin smear was prepared using a drop of blood on a slide. After labelling, slide was air‐dried and fixed in methyl alcohol. Remaining blood was transferred into vacutainer containing anticoagulant (EDTA) and transported to the laboratory for further analysis (Kessell, 2015).

### 2.3 Microscopic examination of blood smear

The fixed thin smears were stained with Giemsa stain at a dilution of 10% in buffer solution and allowed to stay for 20 min followed by washing with tap water to remove extra stain. Then the slides were air dried and examined for the presence of *Anaplasma* inclusion bodies under light microscope.

### 2.4 DNA extraction

Genomic DNA Extraction Kit (Addbio^®^, Korea) was used to extract DNA according to the manufacturer's instructions. Briefly, 20 μl of proteinase K solution was added to 1.5 ml micro‐centrifuge tube containing of whole blood sample (200 μl). Then binding solution (200 μl) was added to the same tube and thoroughly mixed (vortexing for 15 s) followed by incubation at 56°C for 10 min. After incubation, absolute ethanol (200 μl) was added and mixed similarly for 15 s. The lysate was carefully transferred into the upper reservoir of the spin column and centrifuged at 13,000 rpm for 1 min. After discarding the flow through, column was washed two times with 500 μl of washing solution 1 and washing solution 2 by centrifugation at 13,000 rpm for 1 min each time. After washing, column was dried by additional centrifugation at 13,000 rpm for 1 min to remove the residual ethanol of spin column. Finally, spin column was transferred to the new 1.5 ml Eppendorf tube and genomic DNA was eluted with 100 μl of elution buffer by centrifugation at 13,000 rpm for 1 min and stored at –20°C for further analysis.

### 2.5 DNA amplification by PCR

PCR amplifications were carried out using a 2720 thermal cycler (Applied Biosystems, USA). It was performed in a total volume of 20 μl for each reaction using mastermix (10 μl), 10 pmol primer (1 μl for each primer), DNA template (4 μl) and nuclease free water (4 μl). Mastermix includes 20 mM Tris‐HCl (pΗ8.8), 100 mM KCl, 0.2% Triton^®^ X‐100, 4 mM MgCl_2,_ protein stabiliser, sediment, loading dye and 0.5 mM each of dATP, dCTP, dGTP and dTTP. For *Anaplasma* sp. detection, 16SrRNA gene was amplified in an initial denaturation at 95°C for 5 min followed by 40 cycles at 95°C for 1 min (denaturation step), 53°C for 1 min (annealing step) and 72°C for 1 min (extension step) with a final extension step for 72°C for 7 min. After amplification of 16SrRNA gene, only samples positive for 16SrRNA gene were further amplified for msp4 gene to confirm *A. ovis*/ *A. marginale*. Initialy, msp4 gene was amplified with *A. ovis* specific primer in an initial denaturation at 95°C for 5 min followed by 30 cycles at 94°C for 30 s (denaturation step), 60°C for15 s (annealing step) and 68°C for 30 s (extension step) with a final extension step for 68°C for 5 min. After that, only samples negative for *A. ovis* specific primer amplifications were subjected to amplification of msp4 gene with *A. marginale* specific primer in an initial denaturation at 95°C for 5 min followed by 30 cycles at 94°C for 30 s (denaturation step), 58°C for 15 s (annealing step) and 68°C for 30 s (extension step) with a final extension step for 68°C for 5 min. In 16SrRNA gene amplification both positive and negative control and in msp4 gene amplification only negative control were used. The PCR products were analyzed in 1.5% agarose gel in 0.5 TAE buffer and visualised in ethidium bromide and UV transilluminator. The details of primer mentioned in Table 1.

**TABLE 1:** Primers used for amplification of gene fragments of *Anaplasma* organism

Organism	Target gene	Primer name	Oligonucleotide sequence (5′–3′)	Amplicon size (bp)	References
*Anaplasma* sp.	16S rRNA	AE‐F AE‐R	F‐GGTACCYACAGAAGAAGTCC R‐TAGCACTCATCGTTTACAGC	345	Parola et al., 2000
*A. ovis*	msp4	AO‐F AO‐R	F‐TGAAGGGAGCGGGGTCATGGG R‐GGTAATTGCAGCCAGGGACTCT	347	Yousefi et al., 2017
*A. marginale*	msp4	AM‐F AM‐R	F‐CTGAAGGGGGAGTAATGGG R‐GGTAATAGCTGCCAGAGATTCC	344	Yousefi et al., 2017

### 2.6 Purification of PCR products and DNA sequencing

Four PCR products (two from *A. ovis* and two from *A. marginale*) were purified using commercial PCR purification Kit (Addbio^®^, Korea) following the procedures described by the manufacturer. Briefly, 40 μl of PCR product was mixed thoroughly with 200 μl of FADF buffer by vortexing. The mixture was then transferred to a FADF column and centrifuged for 1 min and the flow through was discarded. Wash buffer (750 μl) was added to the column and centrifuged for 1 min. After discarding the flow through the column was centrifuged again for 3 min to dry and placed on a new microcentrifuge tube. Elution buffer (40 μl) containing 10 mM Tris‐HCl (pH 8.5) was added to the column and incubated at room temperature for 2 min. Column was then centrifuged for 2 min to collect the eluted DNA. Sequencing of purified PCR products were done by commercial sequencing company by conventional Sanger sequencing (Addbio^®^, Korea).

### 2.7 Phylogenetic analysis

Once sequences were available, checked initially using BLAST through NCBI (The National Center for Biotechnology Information: http://blast.ncbi.nlm.nih.gov/Blast.cgi) website. After that, we submitted our study sequences in GenBank and received the accession number. These were MN481609.1 and MN481610.1 for *A. ovis* and MN481607.1 and MN481608.1 for *A. marginale*. By the BLASTn homology search and sequences published in GenBank, nucleotide sequences were determined as corresponding to *A. ovis* and *A. marginale*. The multiple alignment and phylogenetic analysis was performed by Mega 10 software by Neighbor joining method (Saitou & Nei, 1987). The evolutionary distances were computed using the p‐distance method by Mega 10 software (Nei & Kumar, 2000). The tree stability was estimated by a boot strap analysis for 1000 replications (Felsenstein, 1985).

### 2.8 Statistical analysis

The obtained information was imported and stored using Microsoft Excel‐2016 to ‘R’ language 3.5.1 version for analysis. A logistic regression was used to compute the odds ratio associated with potential risk factors. The prevalence was expressed in percentage with *p* value for chi‐square test and 95% CI calculated by the modified Wald method using the Graph Pad software Quick Cales. Variables that presented *p* ≤ 0.20 in univariable analysis were considered for including in the multivariable regression model. Forward stepwise selection approach was used to build the final model. Variables with a *p* < 0.05 were considered significant and kept in the final model. The logistic regression analysis was performed using the glmer function from the lme4 package in R version 3.5.1 (R CoreTeam, 2018).

## 3 RESULTS

### 3.1 Prevalence of anaplasmosis in goat

**FIGURE 1:**
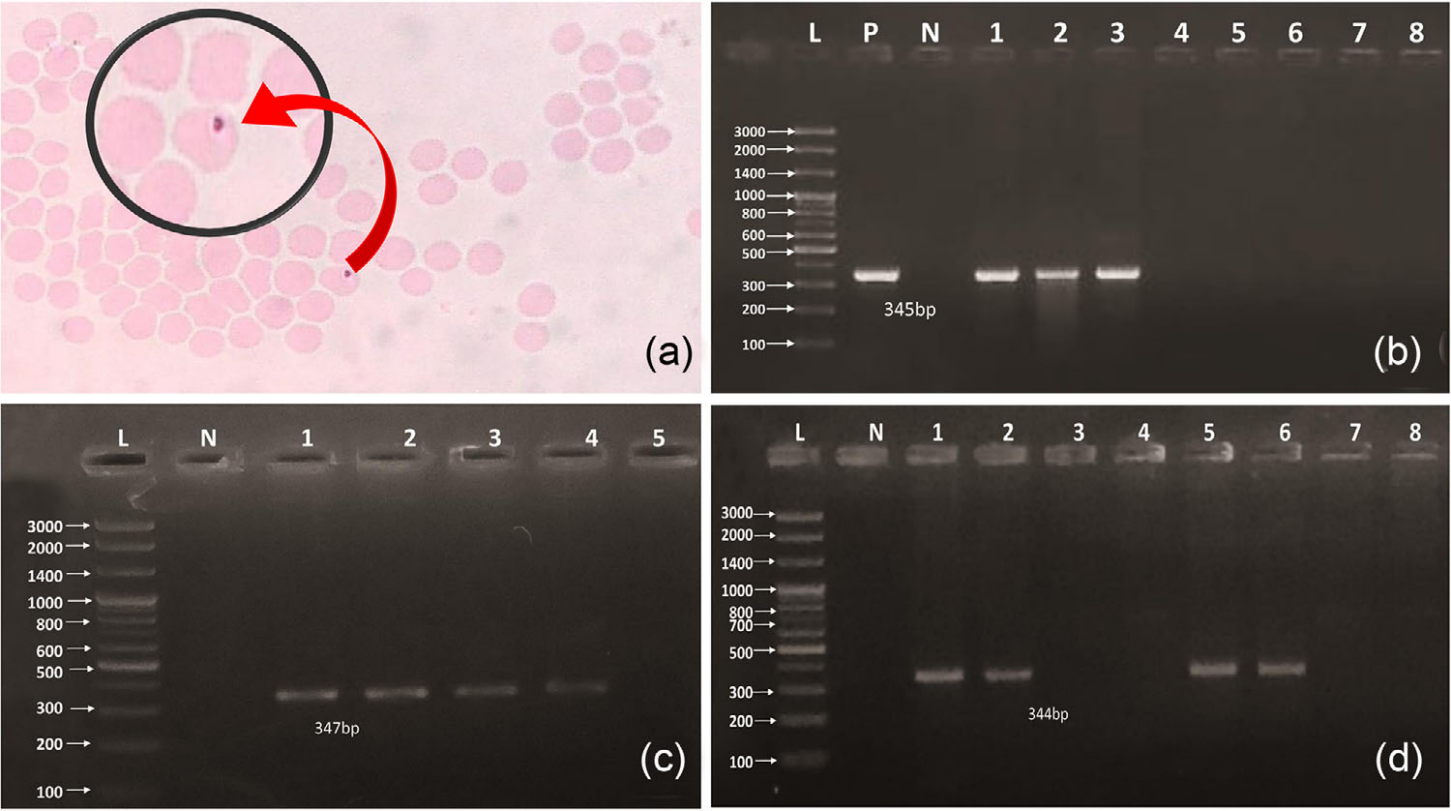
*Anaplasma* inclusion body (magnified circle) within the Red Blood Cell (RBC) (a). Amplification of 16SrRNA gene (345 bp) of *Anaplasma* sp. using genomic DNA extracted from blood of goat. Lane L is for 100 bp plus DNA ladder; Lane P is for positive control and N is for negative control; Lanes 1–8 is suspected samples; Lanes 1–3 having amplicons of 345 bp indicated presence of *Anaplasma* sp. (b). Amplification of msp4 gene specific to *A. ovis* using genomic DNA extracted from blood of goat. Lane L is for 100 bp plus DNA ladder; N is for negative control; Lanes 1–5 is suspected samples; Lanes 1–4 having amplicons of 347 bp indicated presence of *A. ovis* (c). Amplification of msp4 gene specific to *A. marginale* using genomic DNA extracted from blood of goat . Lane L is for 100 bp plus DNA ladder; N is for negative control; Lanes 1–8 is suspected samples; Lanes 1, 2, 5, 6 having amplicons of 344 bp indicated presence of *A. marginale* (d)

Out of 400 whole blood samples, in Giemsa staining technique, 23 (Figure 1a) and in PCR for 16SrRNA gene, 63 samples were found to be positive for *Anaplasma* sp. (345 bp, Figure 1b). All 63 positive samples were further analyzed through PCR for msp4 gene specific to *A. ovis* (347 bp, Figure 1c) and *A. marginale* (344 bp, Figure 1d) and found to be positive as 59 and 4 respectively.

Thus, the prevalences of anaplasmosis were estimated as 5.75% (95% CI: 3.82–8.52) and 15.75% (95% CI: 12.49–19.66) by conventional blood smear and molecular methods (PCR), respectively. The most prevalent infection was *A. ovis* (14.75%; 95% CI: 11.59–18.58) in compare to *A. marginale* (1.0%; 95% CI: 0.01–1.55) among the prevalence of *Anaplasma sp*. (Table 2).

**TABLE 2:** Overall prevalence of anaplasmosis in goat (*N* = 400)

Methods applied	Organism	Positive (*n*)	Prevalence (%)	95% CI
Microscopy (Giemsa stain)	*Anaplasma* sp.	23	5.75	3.82–8.52
Molecular (PCR)	*Anaplasma* sp.	63	15.75	12.49–19.66
	*A. ovis*	59	14.75	11.59–18.58
	*A. marginale*	4	1.00	0.01–1.55

### 3.2 Risk factor associated with anaplasmosis in goats

Different risk factors like season, age, sex, breed, body condition score, coat color, tick infestation, acaricide practice and other management systems (rearing system, grazing, flock size) were considered in univariable logistic regression analysis. Among factors, breed, acaricide uses and tick infestation were found to be significantly (*p* < 0.05) associated with anaplasmosis (Table 3) in goats. No significant relations were observed in the case of season, age, sex, body condition score, coat color and management systems of goats rearing on anaplasma infection in the study area. Factors with *p* ≤0.20 in univariate analysis were further gone for multivariate logistic regression analysis. Tick infestation and uses of acaricides were found as significantly association with anaplasmosis in goat in this study (Table 4).

**TABLE 3:** Univariable logistic regression analysis of risk factors associated with anaplasmosis in goat

Explanatory variable	Co‐variable	Total	+ve	Percentage (95%CI)	OR (95%CI)	*p* Value (χ^2^ test)
Season	Rainy	140	18	12.85 (8.85–20.91)	Reference	0.507
	Summer	133	23	17.29 (11.75–24.68)	1.41 (0.72–2.79)	
	Winter	127	22	17.32 (11.66–24.91)	1.42 (0.72–2.81)	
Rearing system	Backyard	281	43	15.30 (11.54–20.00)	Reference	0.909
	Intensive	68	11	16.17 (9.10–26.87)	1.06 (0.49–2.13)	
	Extensive	51	9	17.64 (9.34–30.48)	1.18 (0.51–2.51)	
Flock size	Large	10	3	30.00 (10.33–60.77)	Reference	0.335
	Medium	35	7	20.00 (9.74–36.19)	0.58 (0.12–3.22)	
	Small	355	53	14.92 (11.58–19.03)	0.37 (0.11–1.94)	
Breed	Bengal goat	262	32	12.21 (8.75–16.77)	Reference	**0.027**
	Jamnapari	17	4	23.52 (9.05–47.77)	2.21 (0.59–6.69)	
	Cross	121	27	22.31(15.77 30.57)	2.06 (1.16–3.63)	
Sex	Female	210	34	16.19 (11.79–21.81)	Reference	0.799
	Male	190	29	15.23 (13.06–24.00)	0.93 (0.54–1.59)	
Age category	Adult	159	25	15.72 (10.83–22.24)	Reference	0.994
	Old	33	5	15.15 (6.17–31.40)	0.95 (0.30–2.54)	
	Young	208	33	15.86 (11.49–21.48)	1.01 (0.57–1.79)	
Body condition	Good	171	20	11.69 (7.63–17.45)	Reference	0.082
	Thin	196	39	19.89 (14.88–26.07)	1.87 (1.05–3.41)	
	Very Thin	33	4	12.12 (4.21–27.93)	1.04 (0.28–3.00)	
Coat color	Black	162	25	15.43 (10.62–21.85)	Reference	0.550
	Brown	115	16	13.91 (8.65–21.51)	0.85 (0.44–1.73)	
	Gray	16	2	12.50 (2.24–37.28)	0.78 (0.11–3.05)	
	Mixed	40	10	25.00 (14.02–40.36)	1.82 (0.76–4.12)	
	White	67	10	14.92 (8.12–25.54)	0.96 (0.41–2.07)	
Grazing	Group	178	34	19.10 (13.97–25.54)	Reference	0.169
	Individual	148	17	11.48 (7.21–17.71)	0.54 (0.28–1.07)	
	No	74	12	16.21 (9.37–26.40)	0.81 (0.38–1.65)	
Acaricide uses	No	337	59	17.50 (13.81–21.94)	Reference	**0.025**
	Yes	63	4	6.34 (2.05–15.67)	0.31 (0.09–0.81)	
Tick infestation	No	381	51	13.38 (10.31–17.19)	Reference	**<0.01**
	Yes	19	12	63.15 (40.94–80.95)	11.09 (4.26–31.05)	

**TABLE 4:** Multivariable logistic regression model output of risk factors associated with anaplasmosis in goat

Explanatory variable	Co‐variable	OR	95% CI	*p* Value (χ^2^ test)
Tick infestation	No	Reference		
	Yes	11.10	4.20–31.62	**<0.01**
Acarcide uses	No	Reference		
	Yes	0.31	0.09–0.84	**0.038**

The prevalence of anaplasmosis was found significantly higher in jamnapari (23.52%; 95% CI: 9.05–47.77) and crossbreed (22.31%; 95%CI: 15.77–30.57) in comparison to Bengal goat (12.21%; 95% CI: 8.75–16.77) (*p* = 0.027) in univariate logistic regression analysis (Table 3).

The study revealed that prevalence of anaplasmosis was higher in goats not using acaricide (17.50%; 95% CI: 13.81–21.94) than acaricide using goats (6.34%; 95% CI: 2.05–15.67). In both univariate and multivariate logistic regression analysis, odd ratio for using acaricides was 0.31 (95% CI: 0.09–0.81 and 0.09–0.84 respectively) (*p* < 0.05) (Tables 3 and 4). This finding indicates that using acaricides is a protective factor for the anaplasmosis in goats.

Regarding tick infestation, the prevalence of anaplasmosis found significantly higher in tick infested goats (63.15%; CI: 40.94–80.95) in compare to non‐tick infested goats (13.38%; CI: 10.31–17.19) (*p* < 0.01). The odd ratio was around 11.0 (95% CI: 4.26–31.05 and 4.20–31.62, respectively) in both univariable and multivariable logistic regression analysis (Tables 3 and 4). The chance of getting anaplasmosis is eleven times higher in tick infested goats than non infested goats.

### 3.3 Phylogenetic analysis

**FIGURE 2:**
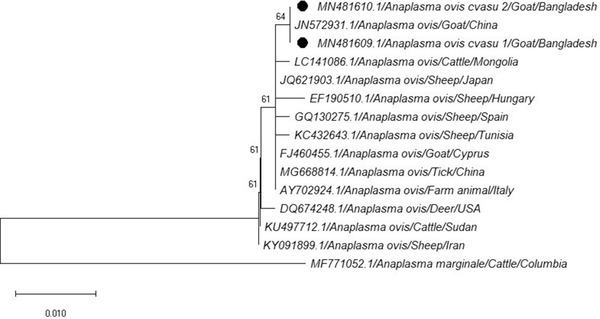
The phylogenetic tree of *A. ovis* obtained from the goat in this study and known *A. ovis* in GenBank using *A. marginale* as related species and outgroups

**FIGURE 3:**
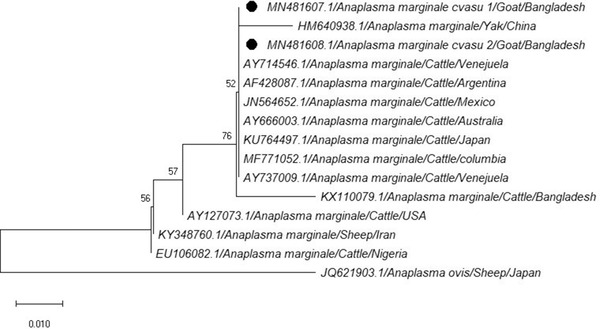
The phylogenetic tree of *A. marginale* obtained from the goat in this study and known *A. marginale* in GenBank using *A. ovis* as related species and outgroups

The multiple alignment and phylogenetic analysis were performed for nucleotide sequences corresponding to *A. ovis* and *A. marginale*. The sequences of *A. ovis* (MN481609.1 and MN481610.1) and *A. marginale* (MN481607.1 and MN481608.1) showed 99% similarity at neocleotide level with isolates of different country that were previously submitted in GenBank database. The tree was constructed using the Neighbour Joining test and Mega 10 software. Sequence of *A. marginale* was used as outgroup for phylogenetic tree of *A. ovis* and vice versa. Topology of *A. ovis* tree shows the close relationship among *A. ovis* (MN481609.1 and MN481610.1) of this study and *A. ovis* from China (JN572931.1) because they positioned within same branch in the same cluster. The nearest relatives to the mentioned cluster are *A. ovis* from Mongolia (LC141086.1), Spain (GQ621903.1), Tunisia (KC432643.1), Cyprus (FJ460455.1), China (MG668814.1) and Italy (AY702924.1) and the lowest similarity was found with the isolate of *A. ovis* from Iran (KY091899.1) (Figure 2). In phylogenetic analysis of *A. marginale*, it is found that, the *s*equences (MN481607.1 and MN481608.1) of this study and *A. marginale* from Brazil (AY714546.1), Argentina (AF428087.1), Mexico (JN564652.1), Australia (AY666003.1) Japan (KU764497.1), Columbia (MF771052.1) and Venezuela (AY737009.1) are closely related. The next nearest relative sequences are *A. marginale* from Bangladesh (KX110079.1) and China (HM640938.1) that are clustered together. *A. marginale* sequence from Nigeria (EU106082.1) was found the lowest similarity with the *A. marginale* sequence of this study (Figure 3).

## 4 DISCUSSIONS

The study was conducted to detect anaplasmosis in goats and estimate their prevalences and associated risk factors of anaplasmosis in goats brought to the Teaching Veterinary Hospital from different areas of Chattogram district. To the best of our knowledge, this study for the first time identified the caprine anaplasmosis with circulating species in Bangladesh. Microscopic examination of thin blood smear is the commonly used tool to detect anaplasmosis in developing countries like Bangladesh. However, with the advancement of modern DNA‐based diagnostic tools, it is now high time to apply state‐of‐the‐art procedures like PCR for comprehensive molecular investigation. Therefore, classical microscopy was complemented by PCR amplification of two different genes of *Anaplasma* sp.

A total of 400 samples were examined by both classical microscopic and molecular technique and estimated prevalences were 5.75% and 15.75%, respectively in goats. The variation in the rate of prevalence in both techniques could be due to high sensitivity of PCR than classical microscopic examination on persistent subclinical infected individuals (Lew et al., 2002). The overall prevalence of anaplasmosis based on classical microscopy (5.75%) found higher than the findings of previous study (2.48%) in goats in Chattogram (Nath et al., 2014) possibly because of differences in diagnostic methods where they only considered clinical signs as diagnostic tool. Again, prevalence rate in this study was lower than that obtained from goats of southern part of India by Rajasokkapan and Selvaraju (2016) (26.15%), goat of Iran by Razmi et al. (2006) (38.92%) and cattle of Chattogram area by Manan (2017) (8.21%). This variation could be due to differences in geographical location of the study, variation in presence of vector ticks in the region and differences in animal species studied. However, a similar observation (5.93%) was reported earlier in Bangladesh though the species was cattle (Samad et al., 1989).

For detection of caprine anaplasmosis, we used molecular technique PCR and overall prevalence was estimated as 15.75% which was lower than the prevalence reported in Iran by Yousefi et al. (2017) and Ahmadi‐Hamedani et al. (2009) where they recorded 34.6% and 63.7% prevalences through PCR, respectively. These differences might be due to different geographical area and climate differences and variation in mechanical and biological vectors. In our study, the prevalence of *A. ovis* (14.75%) found higher than the *A. marginale* (1.00%) which is in agreement with the findings of Yousefi et al. (2017) in Iran where they reported the prevalence of *A. ovis* (34.6%) was higher than *A. marginale* (0.00%). Differences between two species may be due to variation of susceptibility of goats to *A. ovis* and *A. marginale*. However, the prevalence of *A. ovis* (14.75%) of this study was inconsistent with the findings of Yousefi et al. (2017) and Ahmadi Hamedani et al. (2009) where they reported 34.7% and 63.7%, respectively, in Iran. In the case of *A. marginale*, 2.73% prevalence was recorded in goats through PCR in Brazil (da Silva et al., 2018) which is dissimilar with our finding (1.00%). This variation may have been resulted from the differences of geography, climate and absence of potential vectors in this region.

The prevalence of anaplasmosis was found to be higher in winter (17.32%) and in summer (17.29%) than the rainy (12.85%) season though they were not statistically significant. Similar seasonal variation was also observed by previous investigators both in goats and cattle, reported higher incidence in winter season (Mannan, 2017; Nath & Bhuiyan, 2013; Rajasokkapan & Selvaraju, 2016). However, Velusamy et al. (2014) reported that there is no seasonal influence on anaplasmosis. All these variations are thought to be due to changes in macroclimate that is essential for breeding of ticks (Vairamuthu et al., 2012). Moreover, contaminated fomites and some biting insects (flies) are also capable to transmit the disease and probably flies found higher in late rainy to winter seasons.

In univariate and multivariate logistic regression analysis, higher risk of *Anaplasma* infection found in extensive (17.64%) than intensive (16.17%) and backyard farming (15.30%). This finding concurs with previous findings of Angwech et al. (2011) and Wesonga et al. (2017) where they reported that, higher prevalence of infection of tick‐borne diseases found in extensively managed cattle. Supporting this findings, this study also revealed that *Anaplasma* infection was more in goats generally used to graze (19.10%) than non‐grazed (16.21%) animal and which is supported by the findings of Rajasokkapan and Selvaraju (2016) who found that tick‐borne diseases found higher in animal grazed in pasture than non grazed. The extensive system of rearing animal used to graze in pasture has direct effect on tick‐borne diseases as possibility of vectors exposure from nature become higher.

In this study, goats from large flock (30.0%) showed more susceptibe than medium (20.0%) and small flocked goats (14.29%). Though these finding found statistically nonsignificant but supported by Kispotta et al. (2016) and Shahnawaz et al. (2011) where they reported that tick‐borne infection increases with the increases of flock size. This could be due to increase chances of tick infestations through contact and instruments of medication and feeding like syringe and feeder.

Our data showed significant effect of breeds on prevalence of anaplasmosis in goats (*p* = 0.027). Higher infection was recorded in jamnapari (23.52%) followed by crossbreed (22.31%) and black Bengal goats (12.21%), respectively. Similarly, Siddiki et al. (2010) showed lower prevalence of hemoparasite infections in indigenous cattle compared to crossbreed reflected higher exposure rates and impaired acquired immunity in crosses. Our findings were also lined with Belal et al. (2014) who revealed local breeds were relatively resistant to ticks and tick‐borne hemoparasite infections (as compared to exotic and crossbreed animals) and, when infected, were less likely to develop clinical disease.

Even though statistically not significant, there was more anaplasma infection found in female (16.19%) than the male goats (15.23%) which was consistent with Yousefi et al. (2017) in goats and Belal et al. (2014) in cattle. They reported that females were more susceptible to anaplasmosis because of stress and insufficient supply of feed during high demand in pregnancy (Kamani et al., 2010). Besides, hormonal imbalances during milk production and breeding time may causes impaired immunity (Kabir et al., 2011; Kamani et al., 2010; Sajid et al., 2009).

Goats less than 1 year old were found in more risk for anaplasma infection in comparison to other age groups. This observation was statistically nonsignificant and was opposite to the finding reported in India (Rajasokkapan & Selvaraju, 2016). The variation of risk in age category could be due to disproportionate sampling of the study.

Body condition score disclosed nonsignificant association of occurrence of anaplasmosis in goats in this study. As expected, poor (19.89%) body‐conditioned goats are more likely to get anaplasmosis than good (11.69%) body‐conditioned goats which is supported by previous studies (Hamsho et al., 2015; Sitotaw et al., 2014; Wodajnew et al., 2015). This variation might be due to the fact that poor body condition has lower immunity and higher chance to get infection with different organisms like anaplasma.

No significant relation found between coat color and the prevalence of anaplasmosis of goat. However, goats with mixed coat color (25.00%) found more susceptible to anaplasmosis followed by black (15.43%), white (14.92%), brown (13.91%) and gray (12.50%) which was supported by Kispotta et al. (2016) who mentioned that breeds with red or black coat and combination of two or more color have higher risk of infection than those with white coat in regions where biting flies were the insect vector.

This study showed a significant association of anaplasmosis with the use of acaricide in goats. Goats not using acaricides were more likely to be positive for anaplasmosis than acaricide using goats. A similar observation has been reported by Kispotta et al. (2016) in cattle. Another study also supports our statement where stated that cattle and goats receiving no routine veterinary care like acaricides, anthelmintics, antiprotozoals were more prone to haemoparasitic infection than those receiving routine veterinary care (Weny et al., 2017). This difference could be due to the fact that animal with routine acaricide uses have better ability to protect tick infestations.

Availability of vectors is one of the potential risk factors for anaplasma infections (Constable et al., 2017). The final model showed that risk of anaplasmosis was significantly higher in goats having tick than non‐tick infested goat (*p* < 0.05). A similar observation was reported by Costa et al. (2013) and agreement with the observation of Yousefi et al. (2017) and Azmat et al. (2018) where they reported the increased pattern of disease incidence with abundance of tick population. This could be because the most of the haemoparasites are harbored and transmitted by the different species of tick. In the absence of vector ticks, the possible way of anaplasma infection is blood‐sucking arthropods and fomites as mechanical transmission vector (Constable et al., 2017; Dantas‐Torres & Otranto, 2017). This form of mechanical transmission is considered to be the major route of dissemination of bovine anaplasmosis in areas of Central and South America and Africa where tick vectors are merely absent (Abdela et al., 2018).

A phylogenetic tree was constructed by the Neighbour Joining test using the Mega 10 software. The minimum similarity was seen for *A. ovis* with the isolate from Iran (KY091899.1) (Yousefi, 2018) and maximum similarity with the isolate from China (JN572931.1) (Liu et al., 2012). The *A. marginale* phylogenetic tree shows the highest relationships with the isolate from Venezuela (AY737009.1) (Franco et al., 2014) and lowest relationship with the isolate from Nigeria (EU106082.1) (Zivkovic et al., 2007). In topology of *A. ovis* we found that our isolates showed higher tree scale value (long branch length) in their respective clade. This high value indicates that there is some genetic divergence among the isolates.

## 5 CONCLUSION

Anaplasmosis was moderately prevalent in goats of Chattogram region where *A. ovis* and *A. marginale* were exist as both pathogenic and subclinical form. Different breeds of goats, no use of acaricide and presence of ticks on host were found as significant risk factors for anaplasmosis in goats. Sequence analysis revealed that isolates of the study were identical to the isolates reported from countries like Iran, Venezuela, China, Hungary, Japan, Italy, Brazil, Mexico, Australia and the United States. In context of our study, it is highly suggestive for the farmers to rear local breed in compare to exotic and crossbred to prevent the disease. Besides, regular acaricide practices are also recommended to control the tick as well as to control the disease.

## CONFLICT OF INTEREST

The authors declare no conflict of interest.

## ETHICAL STATEMENT

The study was conducted following approval from the Institutional Ethics Committee of Chattogram Veterinary and Animal Sciences University (CVASU), Chattogram, Bangladesh, protocol number: CVASU/Dir(R&E) EC/2020/165(4).

## AUTHOR CONTRIBUTIONS

MMR and MRF reviewed, designed and planed the study. MR was involved in sample collection, laboratory work, data analysis and initially drafted the manuscript. MYEC supervised the whole work. The manuscript was read and approved by each authors prior to submit.

### PEER REVIEW

The peer review history for this article is available at https://publons.com/publon/10.1002/vms3.775

## Data Availability

The data supporting the findings of the study are available within the article and its supplementary material.
